# Dietary intake of branched-chain amino acids in relation to depression, anxiety and psychological distress

**DOI:** 10.1186/s12937-021-00670-z

**Published:** 2021-01-29

**Authors:** Glareh Koochakpoor, Asma Salari-Moghaddam, Ammar Hassanzadeh Keshteli, Hamid Afshar, Ahmad Esmaillzadeh, Peyman Adibi

**Affiliations:** 1 Maragheh University of Medical Sciences, Maragheh, Iran; 2 Department of Community Nutrition, School of Nutritional Sciences and Dietetics, Tehran University of Medical Sciences, P.O. Box 14155-6117, Tehran, Iran; 3 Department of Medicine, University of Alberta, Edmonton, Alberta Canada; 4 Integrative Functional Gastroenterology Research Center, Isfahan University of Medical Sciences, Isfahan, Iran; 5 Department of Psychiatry, Psychosomatic Research Center, Isfahan University of Medical Sciences, Isfahan, Iran; 6 Obesity and Eating Habits Research Center, Endocrinology and Metabolism Molecular -Cellular Sciences Institute, Tehran University of Medical Sciences, Tehran, Iran; 7 Department of Community Nutrition, School of Nutrition and Food Science, Isfahan University of Medical Sciences, Isfahan, Iran

**Keywords:** Dietary BCAA, Depression, Anxiety, Psychological distress

## Abstract

**Background:**

There is no previous study that examined the association between branched-chain amino acids (BCAAs) intake and odds of psychological disorders. The aim of this study was to investigate the association between dietary BCAAs and odds of psychological disorders including depression, anxiety, and psychological distress in a large sample of Iranian adults.

**Methods:**

In this cross-sectional study on 3175 Iranian adults aged 18–55 years, a validated food frequency questionnaire was used to assess dietary intakes. BCAAs intake was computed by summing up the amount of valine, leucine, and isoleucine intake from all food items in the questionnaire. Psychological health was examined through the use of Iranian validated version of the Hospital Anxiety and Depression Scale (HADS). Psychological distress was assessed using General Health Questionnaire (GHQ). For depression and anxiety, scores of 8 or more on either subscale were considered as psychological disorders and scores of 0–7 were defined as “normal”. In terms of psychological distress, the score of 4 or more was defined as psychological distress.

**Results:**

Mean age of study participants was 36.2 ± 7.8 years. Overall, 26.4% (*n* = 837) of study subjects had depression, 11.9% (*n* = 378) had anxiety and 20.9% (*n* = 665) were affected by psychological distress. After controlling for potential confounders, participants in the highest tertile of total BCAAs intake had lower odds of depression (OR: 0.76; 95% CI: 0.60–0.96) and anxiety (OR: 0.66; 95% CI: 0.47–0.91) compared with those in the lowest tertile. Participants in the top tertile of valine intake had a lower odds of depression (OR: 0.76; 95% CI: 0.60–0.96) and anxiety (OR: 0.65; 95% CI: 0.47–0.90) compared with those in the bottom tertile. A significant inverse association was also seen between leucine intake and depression (OR: 0.77; 95% CI: 0.61–0.98) and anxiety (OR: 0.66; 95% CI: 0.47–0.91). In addition, a significant inverse association was observed between isoleucine intake and odds of depression (OR: 0.75; 95% CI: 0.59–0.95) and anxiety (OR: 0.62; 95% CI: 0.45–0.86). There was no significant association between isoleucine intake and odds of psychological distress.

**Conclusion:**

Evidence indicating an inverse association between dietary intake of BCAAs and odds of depression and anxiety was found. Prospective studies are required to confirm these findings.

## Introduction

Depression, anxiety, and psychological distress are among the most common psychiatric disorders affecting emotional and behavioral control [1]. They can substantially deteriorate one’s functioning in the family and workplace [2]. High prevalence of psychological disorders, both in developed and developing countries, imposes heavy social and economic burdens on health care systems [3]. Due to high prevalence of these conditions, early onset, increased mortality, high medical costs and disability, finding modifiable risk factors are of high priority.

Prescribed medicines are not effective enough in the treatment of common mental disorders. Among those receiving these treatments, only 60% reported improved symptoms [4]. Therefore, there is a great need for adjunctive treatment. In a meta-analysis, dietary interventions significantly reduced depressive symptoms [5]. Chemical imbalance in brain neurotransmitters is often cited as an explanation for psychiatric disorders [6]. In addition, it is well-documented that adequate amounts of nutrients are necessary for the production of neurotransmitters [7]. Therefore, recent studies have focused on the role of dietary intakes in the management of psychological disorders. Several studies showed that aromatic amino acids such as tryptophan, tyrosine, and phenylalanine might be useful in treating depression by producing neurotransmitters [8, 9]. Branched-chain amino acids (BCAAs) compete with aromatic amino acids to transfer from blood-brain barrier. Therefore, the higher BCAAs concentrations in the blood, the lower aromatic amino acids will be observed in the brain [10]. Therefore, it is possible that reductions in brain concentrations of aromatic amino acids will subsequently reduce the synthesis and the release of neurotransmitters associated with aromatic amino acids. Baranyi et al. conducted a case-control study on 71 in-patients with major depression and 48 healthy controls [11]. They reported that the concentrations of BCAAs are significantly decreased in patients with major depression in comparison with healthy subjects. Previous studies have shown a direct association between dietary intakes of BCAAs and their serum concentrations [12, 13]. Milk, red meat, poultry, and dairy products are the main dietary sources of BCAAs [14]. The association between food sources of BCAAs and depression was also found in earlier publications [15]. There is no study examining the association between dietary intake of BCAAs and odds of psychological disorders. The purpose of this study was, therefore, to examine the association between dietary BCAAs and odds of psychological disorders including depression, anxiety, and psychological distress in a large sample of Iranian adults.

## Methods and materials

### Participants

The present cross-sectional study was conducted within the framework of the SEPAHAN (Study on the Epidemiology of Psychological, Alimentary Health and Nutrition) project, a cross-sectional study that investigated the prevalence of functional gastrointestinal disorders (FGIDs) and their relationship to lifestyle factors. Detailed information about data collection methods in SEPAHAN project has been published previously [16]. In brief, this study was performed among Iranian general adults working in 50 different healthcare centers affiliated to Isfahan University of Medical Sciences (IUMS) across Isfahan province. In this project, data were collected in two main phases between April 2010 and May 2010. A questionnaire that contained information on demographic and dietary data was sent to 10,087 participants and 8691 subjects returned the completed questionnaire (response rate of 86.16%), in the first step. At the second phase, information on psychological health was collected and 6239 subjects returned the completed questionnaire. Some participants who took part in the second phase did not participate in the first phase. In addition, some participants in both phases had not written their name or national identification code on at least one of the questionnaires. This made us unable to match the data for all subjects participated in both phases. Then, 4763 questionnaires in the second phase were matched with their equivalent questionnaires in the first phase. In the current study, subjects who had total daily energy intakes outside the range of 800–4200 kcal/d were excluded due to under- or over-reporting of energy intake [17]. In addition, those who had missing data on any relevant variable were excluded. Individuals with anti-depressant use were also excluded from the current analysis. Therefore, data from 3175 subjects, for whom complete information about both dietary intakes and psychological profile were available, were included in the current analysis. All participants provided written informed consent forms. The study protocol was ethically approved by the Regional Bioethics Committee of Isfahan University of Medical Sciences (#189069, #189082, and #189086). This cross-sectional study was reported in according to ESTROBE guideline.

### Dietary intakes assessment

Dietary data were collected using a Willett-format Dish-based 106-item Semi-quantitative Food Frequency Questionnaire (DS-FFQ), which was designed and validated specifically for Iranian adults. Detailed information about the design, foods included as well as the face validity of this questionnaire has been reported elsewhere [18]. Briefly, the questionnaire contained five categories of foods and dishes: [1] mixed dishes (cooked or canned, 29 items) [2]; carbohydrate-based foods (different types of bread, cakes, biscuits and potato, 10 items) [3]; dairy products (dairies, butter and cream, 9 items) [4]; fruits and vegetables (22 items); and [5] miscellaneous food items and beverages (including sweets, fast foods, nuts, desserts and beverages, 36 items). For each food item, a commonly consumed portion size was defined. Participants were asked to report their dietary intakes of foods and mixed dishes based on nine multiple choice frequency response categories varying from “never or less than once a month” to “12 or more times per day”. The frequency response categories for the food list varied from 6 to 9 choices. For foods consumed infrequently, the high-frequency categories were omitted, while for common foods with a high consumption, the number of multiple choice categories increased. For instance, the frequency response for tuna consumption included six categories, as follows: never or less than once/month, 1–3 times/month, 1 time per week, 2–4 times/week, 5–6 times/week, 1–2 times/day; and for tea consumption that is highly prevalent among Iranians, the frequency response included nine categories, as follows: never or less than 1 cup/month, 1–3 cups/month, 1–3 cups/week, 4–6 cups/week, 1 cup/day, 2–4 cups/day, 5–7 cups/day, 8–11 cups/day, ≥12 cups/day). Finally, to convert the food items into grams, the amount of each portion size was computed based on the booklet of “household measures” [19], and then computed the amount of intake by considering the frequency of consumption of each food item. Because the Iranian food composition table is incomplete, the United States Department of Agriculture (USDA) food composition table was used to analyze foods and beverages; however, in the dataset of USDA in the software, some traditional foods and beverages were modified based on the Iranian food composition table, which were not listed in the USDA food composition table. This means that for almost 98% of foods in the FFQ, the USDA database was used. For some food items that were not available there (for example Iranian local breads like Lavash and Barbari) and the nutrient composition of these foods were available in Iranian food composition table [20], these foods were added to the database of the software. Nutrient intakes for each participant was calculated using the USDA food composition database that was modified for Iranian foods. Milk, red meat, poultry, and dairy products are the major dietary sources of BCAAs [14]. The amount of valine, leucine, and isoleucine in 100 g of these foods was calculated and then total BCAAs intake was computed by summing up the amount of valine, leucine, and isoleucine intake.

The validity of DS-FFQ was examined in a subgroup of 200 randomly selected participants of the SEPAHAN project. All participants in the validation study completed the DS-FFQ at study baseline and 6 months later. During this validation study, participants provided three detailed dietary records that were used as the gold standard. As shown in earlier studies, it seems that this questionnaire provides reasonably valid measures of long-term dietary intakes [18].

### Assessment of the psychological profile

The Iranian validated version of Hospital Anxiety and Depression Scale (HADS) was used to screen for anxiety and depression [21]. HADS is a brief and useful questionnaire to assess psychological disorders and symptom severity of depression and anxiety disorders. The HADS contains 14 items and include two subscales: anxiety and depression. Each item includes a four-point scale; higher scores indicate an elevated level of anxious and depressive symptomatology. Maximum score is 21 for anxiety and depression. Scores of 8 or more on either subscale were considered as psychological disorders and scores of 0–7 were defined as “normal” in the current study. The convergent validation of translated version of HADS questionnaire was examined in 167 Iranian adults using the correlation of each item with its hypothesized scale. Pearson’s correlation coefficients varied from 0.47 to 0.83 (*P* < 0.0001) for anxiety subscale and from 0.48 to 0.86 (*P* < 0.0001) for depression subscale, indicating that the questionnaire provides relatively valid measures of psychological health [21]. The Iranian validated version of General Health Questionnaire (GHQ) with 12-items was used to assess psychological distress [22]. GHQ-12 is a brief, simple, easy-to-complete instrument for measuring current and primary mental health that asks the respondents whether they have experienced a particular symptom of psychological distress or a change in their behavior recently. Each item consists of a four-point scale (less than usual, no more than usual, rather more than usual, or much more than usual). In this study, the bimodal (0–0–1-1) scoring method was used. This gives scores ranging from 0 to 12. Higher scores indicate a greater degree of psychological distress. In the current study, the score of 4 or more was defined as having psychological distress [23]. The convergent validity of GHQ-12 was examined in 748 Iranian young people. Significant inverse correlation was seen between the GHQ-12 and global quality of life scores (*r* = − 0.56, *P* < 0.0001) [22].

### Assessment of other variables

Required information on other variables including age, sex, marital status, smoking status, education, and chronic conditions (diabetes, asthma, colitis, stroke, myocardial infarction, heart failure, and cancers) and antidepressant and supplements (vitamins, minerals, calcium and iron) was obtained from demographic and medical history questionnaires. Physical activity was assessed using the General Practice Physical Activity Questionnaire (GPPAQ) [24], and participants were classified into two categories: physically active (≥1 h/week) and physically inactive (< 1 h/week). Anthropometric measures including weight, height, and waist circumference were assessed using a self-administered questionnaire. The validity of self-reported values of weight, height, and waist circumferences (WC) was examined in a pilot study on 200 participants from the same population. In the validation study, self-reported values of anthropometric indices were compared with actually measured values. The correlation coefficients for self-reported weight, height, and WC versus corresponding measured values were 0.95 (*P* < 0.001), 0.83 (*P* < 0.001), and 0.60 (*P* < 0.001), respectively. Body Mass Index (BMI) was calculated by dividing weight (kg) to height (m^2^). The correlation coefficient for computed BMI from self-reported values, and the one from measured values was 0.70 (*P* < 0.001) [25].

### Statistical analysis

General characteristics of study participants across tertiles of BCAAs were expressed as means ±SDs for continuous variables and percentages for categorical variables. Dietary intakes of study participants across tertiles of BCAAs were compared using analysis of variance (ANOVA). Binary logistic regression was used to estimate ORs and 95% CIs for the presence of psychological disorders across tertiles of BCAAs in crude and multivariable-adjusted models. Age (continuous), sex (male/female), total energy intake (continuous), marital status (married/single), education (diploma or under-diploma/university graduate), vitamin supplements use (yes/no), smoking (non-smoker/former smokers and current smokers), physical activity (< 1 h/week/≥1 h/week), presence of chronic conditions (yes/no), dietary intakes of omega 3, fiber, group B vitamins, fruits, vegetables, and BMI were controlled for in the multivariable-adjusted model. P for trends was determined by considering tertiles of BCAAs intake as ordinal variables in the logistic regression analysis. All statistical analyses were done using the Statistical Package for Social Sciences (version 20; SPSS Inc.). *P* < 0.05 was considered as statistically significant.

## Results

Overall, 26.4% (*n* = 837) of study subjects had depression, 11.9% (*n* = 378) had anxiety and 20.9% (*n* = 665) were affected by psychological distress. The mean intake of total BCAAs, valine, leucine, and isoleucine was 11.18 ± 4.46, 3.41 ± 1.33, 4.85 ± 1.93, and 2.91 ± 1.19, respectively. The correlation coefficients between BCAAs and depression, anxiety, and psychological distress were − 0.65, − 0.049, and − 0.051, respectively. Baseline characteristics of study participants across tertiles of BCAAs, valine, leucine, and isoleucine are presented in Table 1. Participants in the top tertile of BCAAs intake were more likely to be physically active, current smoker, had chronic disease and higher BMI and less likely to be female compared to those in the bottom tertile. Those in the highest tertile of valine intake were more likely to be older, physically active, current smoker, had chronic diseases and higher BMI and less likely to be female compared with those in the lowest tertile. Participants in the highest tertile of leucine intake were more likely to be physically active, current smoker, had chronic diseases and higher BMI and less likely to be female compared with those in the lowest tertile. In terms of isoleucine, participants in the top tertile of isoleucine intake were more likely to be physically active, current smoker, had chronic diseases and higher BMI compared with those in the bottom tertile. No other significant differences were found in terms of other variables.

**Table 1:** General characteristics of study participants across categories of BCAAs^a^

	BCAAs		P-value^b^	Valine		P-value^b^	Leucine		P-value^b^	Isoleucine		P-value^b^
	T_1_	T_3_		T_1_	T_3_		T_1_	T_3_		T_1_	T_3_	
Age (y)	35.9 ± 7.6	35.9 ± 7.6	0.05	35.8 ± 7.5	36.6 ± 8.1	0.005	35.9 ± 7.6	36.6 ± 8.2	0.06	35.9 ± 7.6	36.6 ± 8.2	0.06
BMI (kg/m^2^)	24.5 ± 3.7	35.9 ± 7.6	0.001	24.5 ± 3.7	35.1 ± 3.8	0.001	24.5 ± 3.6	25.1 ± 3.8	0.001	24.5 ± 3.7	25.1 ± 3.8	0.002
Female (%)	58.2	54.2	0.02	57.8	54.9	0.07	58.0	54.4	0.03	57.6	54.8	0.05
Married (%)	80.3	80.5	0.10	80.2	80.5	0.14	80.1	80.5	0.06	80.5	80.1	0.05
University graduated (%)	63.6	62.3	0.81	63.3	62.3	0.88	63.3	62.2	0.83	62.5	62.2	0.68
Physically active (≥1 h/week)(%)	12.2	16.0	0.006	12.1	16.0	0.005	12.3	16.2	0.002	12.0	16.2	0.003
Current smokers (%)	11.0	14.0	0.01	11.0	15.2	0.01	10.9	15.5	0.007	10.9	15.0	0.01
Chronic diseases (%)	3.1	5.6	0.01	3.0	5.5	0.01	3.1	5.5	0.01	3.4	5.6	0.01
Dietary supplement use (%)	28.6	29.5	0.89	28.7	29.8	0.85	28.6	29.1	0.86	28.4	30.0	0.70

^**a**^**All values are mean ± standard deviation (SD), unless indicated**

^**b**^**ANOVA for continuous variables and chi-squared test for categorical variables**

Dietary intakes of study participants across tertiles of valine, leucine and isoleucine intake are shown in **Table** **2**. Compared with those in the lowest tertile of valine and isoleucine intake, those in the highest tertile had higher intakes of energy, protein, fat, vitamin B2, vitamin B5, vitamin B6, vitamin B12, calcium, zinc and tryptophan and lower intakes of carbohydrates, dietary fiber, vitamin B1, vitamin B3, folate and iron. In terms of leucine intake, participants in the highest tertile of leucine intake had higher intakes of energy, protein, fat, vitamin B5, vitamin B6, vitamin B12, calcium, zinc and tryptophan and lower intakes of carbohydrates, dietary fiber, vitamin B1, vitamin B2, vitamin B3, folate and iron.

**Table 2:** Dietary intakes of study participants across categories of valine, leucine and isoleucine intake^a^

	Valine intake			P-value^b^	Leucine intake			P-value^b^	Isoleucine intake			P-value^b^
	T_1_	T_2_	T_3_		T_1_	T_2_	T_3_		T_1_	T_2_	T_3_	
Energy (kcal/d)	2458.0 ± 873	2172.2 ± 783	2531.3 ± 770	< 0.001	2460.4 ± 865	2170.8 ± 791	2531.3 ± 770	< 0.001	2472.4 ± 861	2180.0 ± 800	2510.0 ± 775	< 0.001
Protein (g/d)	81.7 ± 32	79.4 ± 29	104.5 ± 32	< 0.001	81.7 ± 32	79.11 ± 29	104.9 ± 32	< 0.001	81.8 ± 31	79.3 ± 29	104.5 ± 32	< 0.001
Fat (g/d)	91.7 ± 34	92.8 ± 35	111.7 ± 36	< 0.001	91.8 ± 34	92.6 ± 35	111.7 ± 37	< 0.001	92.2 ± 33	92.4 ± 35	111.6 ± 37	< 0.001
Carbohydrate (g/d)	334.0 ± 129	263.1 ± 105	286.1 ± 101	< 0.001	334.4 ± 128	263.5 ± 106	285.4 ± 101	< 0.001	336.5 ± 128	266.0 ± 107	280.7 ± 99	< 0.001
Dietary fiber (g/d)	23.7 ± 9	20.8 ± 9	23.1 ± 9	< 0.001	23.7 ± 9	20.79 ± 9.3	23.1 ± 9.6	< 0.001	23.8 ± 9	21.0 ± 9	22.8 ± 9	< 0.001
Vitamin B1 (mg/d)	2.1 ± 1	1.5 ± 0.7	1.7 ± 0.6	< 0.001	2.1 ± 1	1.5 ± 0.7	1.7 ± 0.6	< 0.001	2.2 ± 1	1.6 ± 0.7	1.7 ± 0.6	< 0.001
Vitamin B2 (mg/d)	1.7 ± 0.7	1.6 ± 0.6	2.2 ± 0.7	< 0.001	2.7 ± 0.7	1.6 ± 0.6	2.1 ± 0.7	< 0.001	1.7 ± 0.7	1.6 ± 0.6	2.1 ± 0.7	< 0.001
Vitamin B3 (mg/d)	27.3 ± 12	22.1 ± 9	25.7 ± 9	< 0.001	27.2 ± 12	21.9 ± 9	26 ± 9.8	< 0.001	27.2 ± 11	22 ± 9	25.9 ± 9	< 0.001
Vitamin B5 (mg/d)	5.8 ± 2	5.6 ± 2	7.1 ± 2	< 0.001	5.9 ± 2	5.6 ± 1	7 ± 2	< 0.001	5.9 ± 2	5.7 ± 1	6.9 ± 1	< 0.001
Vitamin B6 (mg/d)	1.8 ± 0.6	1.8 ± 0.7	2.2 ± 0.7	< 0.001	1.8 ± 0.6	1.8 ± 0.7	2.2 ± 0.7	< 0.001	1.8 ± 0.6	1.8 ± 0.7	2.2 ± 0.7	< 0.001
Vitamin B12 (μg/d)	2.3 ± 1	2.7 ± 1	3.8 ± 1	< 0.001	2.3 ± 1	2.7 ± 1	3.8 ± 1	< 0.001	2.35 ± 1	2.7 ± 1	3.7 ± 1	< 0.001
Folate (μg/d)	636.6 ± 280	518.9 ± 205	570.3 ± 204	< 0.001	637.3 ± 279	517.5 ± 204	570.5 ± 205	< 0.001	642.2 ± 279	523.2 ± 208	560.6 ± 200	< 0.001
Fe (mg/d)	19.7 ± 8	15.7 ± 6	17.4 ± 6	< 0.001	19.7 ± 8	15.7 ± 6.5	17.4 ± 6	< 0.001	19.8 ± 8.8	15.8 ± 6.6	17.29 ± 6	< 0.001
Ca (mg/d)	1020.6 ± 665	805.7 ± 399	1113.5 ± 496	< 0.001	1028.1 ± 662	817.3 ± 412	1095.9 ± 497	< 0.001	1043 ± 662	830.7 ± 418	1067.6 ± 501	< 0.001
Zn (mg/d)	10.2 ± 3	10.1 ± 3	12.8 ± 4	< 0.001	10.2 ± 3	10.1 ± 3	12.8 ± 3	< 0.001	10.3 ± 3.7	10.2 ± 3	12.7 ± 3	< 0.001
Tryptophan (μmol/l)	0.5 ± 0.1	0.6 ± 0.2	0.9 ± 0.3	< 0.001	0.5 ± 0.1	0.64 ± 0.2	0.92 ± 0.2	< 0.001	0.5 ± 0.1	0.6 ± 0.2	0.9 ± 0.2	< 0.001

^**a**^**Data are mean ± standard deviation (SD)**

^**b**^**Obtained from ANOVA**

Crude and multivariable-adjusted odds ratios (ORs) and 95% confidence intervals (95% CIs) for depression, anxiety and psychological distress across tertiles of BCAAs intake are presented in Table 3. After controlling for potential confounders, participants in the highest tertile of BCAAs had lower odds of depression compared with those in the lowest tertile (OR:0.76; 95% CI: 0.60–0.96). Participants in the highest tertile of BCAAs had 34% lower odds of anxiety compared with those in the lowest tertile (OR: 0.66; 95% CI: 0.47–0.91). No significant association was found between BCAAs and odds of psychological distress (OR: 0.84; 95% CI: 0.65–1.09). When the analysis was confined among those taking antidepressants, no significant association was found between BCAAs and odds of depression (OR: 1.41; 95% CI: 0.54–3.70), anxiety (OR: 1.33; 95% CI: 0.51–3.48), and psychological distress (OR: 0.68; 95% CI: 0.27–1.70).

**Table 3:** Crude and multivariable-adjusted ORs and 95% CIs for psychological disorders across tertiles of BCAAs intake^a^

	BCAAs intake			
	T_1_	T_2_	T_3_	P-trend
**Depression**				
**Cases, n**	297	271	269	
Crude	1.00	1.06 (0.88–1.29)	0.81 (0.67–0.99)	0.04
Model I	1.00	1.02 (0.82–1.25)	0.82 (0.66–1.01)	0.07
Model II	1.00	1.02 (0.83–1.27)	0.79 (0.63–0.98)	0.04
Model III	1.00	1.01 (0.81–1.26)	0.77 (0.61–0.97)	0.03
Model IV	1.00	1.01 (0.80–1.26)	0.76 (0.60–0.96)	0.02
**Anxiety**				
**Cases, n**	146	125	107	
Crude	1.00	0.86 (0.66–1.11)	0.71 (0.55–0.93)	0.01
Model I	1.00	0.77 (0.59–1.02)	0.69 (0.52–0.91)	0.01
Model II	1.00	0.78 (0.59–1.03)	0.65 (0.48–0.87)	0.004
Model III	1.00	0.81 (0.61–1.09)	0.68 (0.50–0.93)	0.02
Model IV	1.00	0.81 (0.60–1.09)	0.66 (0.47–0.91)	0.011
**Psychological distress**				
**Cases, n**	217	232	216	
Crude	1.00	1.01 (0.82–1.24)	0.80 (0.64–0.99)	0.04
Model I	1.00	1.03 (0.83–1.29)	0.82 (0.65–1.03)	0.09
Model II	1.00	1.01 (0.81–1.27)	0.81 (0.65–1.03)	0.09
Model III	1.00	1.07 (0.84–1.35)	0.86 (0.67–1.10)	0.24
Model IV	1.00	1.06 (0.83–1.34)	0.84 (0.65–1.09)	0.20

^**a**^**Data are OR (95% CI)**

**Model I: adjusted for age, sex and energy intake**

**Model II: additionally, adjusted for marital status, education, vitamin supplements use, smoking status, physical activity and chronic conditions**

**Model III: additionally, adjusted for dietary fiber, omega 3, vitamin B1, vitamin B2, vitamin B3, vitamin B6, vitamin B12, folate, fruits and vegetables**

**Model IV: additionally, adjusted for BMI**

Crude and multivariable-adjusted ORs and 95% CIs for depression, anxiety and psychological distress across tertiles of valine, leucine and isoleucine are presented in Table 4. After adjustment for potential confounders, participants in the top tertile of valine intake had a lower odds of depression (OR: 0.76; 95% CI: 0.60–0.96) and anxiety (OR: 0.65; 95% CI: 0.47–0.90) compared with those in the lowest tertile. No significant association was found between valine intake and odds of psychological distress (OR: 0.87; 95% CI: 0.67–1.12). A significant inverse association was seen between leucine intake and depression (OR: 0.77; 95% CI: 0.61–0.98) and anxiety (OR: 0.66; 95% CI: 0.47–0.91). No significant association was observed between leucine intake and odds of psychological distress. In the fully adjusted model, a significant inverse association was found between isoleucine intake and odds of depression (OR: 0.75; 95% CI: 0.59–0.95) and anxiety (OR: 0.62; 95% CI: 0.45–0.86). There was no significant association between isoleucine intake and odds of psychological distress.

**Table 4:** Crude and multivariable-adjusted ORs and 95% CIs for psychological disorders across categories of valine, leucine and isoleucine intake^a^

	Valine intake		P-trend	Leucine intake		P-trend	Isoleucine intake		P-trend
	T_1_	T_3_		T_1_	T_3_		T_1_	T_3_	
**Depression**									
**Cases, n**	290	254		288	254		288	255	
Crude	1.00	0.82 (0.67–1.00)	0.05	1.00	0.82 (0.68–1.00)	0.06	1.00	0.83 (0.68–1.01)	0.06
Model I	1.00	0.82 (0.66–1.01)	0.07	1.00	0.83 (0.67–1.03)	0.09	1.00	0.81 (0.66–1.00)	0.06
Model II	1.00	0.79 (0.64–0.98)	0.04	1.00	0.80 (0.65–1.00)	0.05	1.00	0.79 (0.63–0.98)	0.03
Model III	1.00	0.77 (0.61–0.98)	0.03	1.00	0.78 (0.62–0.99)	0.04	1.00	0.77 (0.61–0.97)	0.03
Model IV	1.00	0.76 (0.60–0.96)	0.02	1.00	0.77 (0.61–0.98)	0.03	1.00	0.75 (0.59–0.95)	0.02
**Anxiety**									
**Cases, n**	144	109		143	108		147	106	
Crude	1.00	0.72 (0.55–0.93)	0.01	1.00	0.71 (0.54–0.93)	0.01	1.00	0.67 (0.51–0.88)	0.004
Model I	1.00	0.69 (0.51–0.91)	0.01	1.00	0.69 (0.51–0.91)	0.01	1.00	0.64 (0.48–0.85)	0.002
Model II	1.00	0.65 (0.48–0.87)	0.004	1.00	0.65 (0.48–0.87)	0.004	1.00	0.61 (0.46–0.83)	0.001
Model III	1.00	0.68 (0.50–0.93)	0.02	1.00	0.68 (0.50–0.94)	0.02	1.00	0.64 (0.47–0.88)	0.006
Model IV	1.00	0.65 (0.47–0.90)	0.009	1.00	0.66 (0.47–0.91)	0.01	1.00	0.62 (0.45–0.86)	0.004
**Psychological distress**									
**Cases, n**	232	204		232	200		233	198	
Crude	1.00	0.83 (0.68–1.02)	0.07	1.00	0.80 (0.65–0.99)	0.04	1.00	0.79 (0.64–0.97)	0.03
Model I	1.00	0.84 (0.67–1.05)	0.14	1.00	0.82 (0.66–1.03)	0.10	1.00	0.80 (0.64–1.01)	0.07
Model II	1.00	0.84 (0.67–1.06)	0.15	1.00	0.82 (0.65–1.04)	0.11	1.00	0.81 (0.64–1.02)	0.08
Model III	1.00	0.89 (0.69–1.14)	0.36	1.00	0.87 (0.68–1.18)	0.27	1.00	0.85 (0.66–1.09)	0.20
Model IV	1.00	0.87 (0.67–1.12)	0.29	1.00	0.85 (0.66–1.10)	0.22	1.00	0.82 (0.64–1.07)	0.14

^**a**^**Data are OR (95% CI)**

**Model I: adjusted for age, sex and energy intake**

**Model II: additionally, adjusted for marital status, education, vitamin supplements use, smoking status, physical activity and chronic conditions**

**Model III: additionally, adjusted for dietary fiber, omega 3, vitamin B1, vitamin B2, vitamin B3, vitamin B6, vitamin B12, folate, fruits and vegetables**

**Model IV: additionally, adjusted for BMI**

## Discussion

In the current study, total BCAAs intake were associated with lower odds of depression and anxiety. Furthermore, higher intake of valine, leucine and isoleucine was associated with reduced odds of depression and anxiety. No significant association was found between total BCAAs, valine, leucine and isoleucine intake and odds of psychological distress. This is the first study examining the association between dietary BCAAs and odds of psychological disorders.

Psychological disorders are among the main leading causes of mortality and morbidity in the world [26]. Diet, as a modifiable risk factor, has attracted a huge attention for management of psychological disorders [27]. In this study, a significant inverse association was found between BCAAs intake and odds of depression and anxiety; however, there was no significant association between BCAAs intake and odds of psychological distress. Given the role of BCAAs in competing with aromatic amino acids [10], it is expected that BCAAs intake might be associated with decreased odds of psychological disorders. Previous cross-sectional study reported that BCAAs were significantly decreased in patients with major depression compared with healthy subjects [11]. In another previous study, the sum of the competing amino acids valine, isoleucine, leucine, tyrosine and phenylalanine also significantly declined in patients with interferon-α induced depression [28].

In an experimental study on rats, dietary supplementation of BCAAs reduces exploratory behavior and increases anxiety [29]. These findings suggest that increased BCAAs levels may influence psychological health. One possible mechanism for behavioral changes in rats was that BCAAs compete with AAAs to transfer from blood-brain barrier [10, 30]. Therefore, an increase in BCAAs would be associated with a decrease in AAAs and, in turn, decrease in their derived neurotransmitters (serotonin, norepinephrine and dopamine) in the rat brain. However, it seems that the reduced AAAs availability in the brain is not sufficient to have an impact on transmitter levels or synaptic function in humans [31]. Because tyrosine hydroxylase, the rate-limiting enzyme in the generation of norepinephrine and dopamine, is close to full saturation with the tyrosine substrate [32, 33], therefore, changes in the availability do not alter the net biosynthetic capability. Reducing the risk of depression and anxiety observed in the current study might lead to an alternative hypothesis that activation of mTor by BCAAs might be associated with a reduction of depression and anxiety. Antidepressants such as ketamine, sertraline, fluoxetine and methylphenidate also induced mTor activity [34, 35]. In contrast, inhibition of mTor by rapamycin reverses the antidepressant effects of ketamine in patients with depression [36]. In addition, one study reported that exposure to chronic stress reduced mTor phosphorylation levels and caused specific brain abnormalities in signaling pathway [37]. Therefore, it is possible that mTor is activated by BCAAs intake, resulting in reduce the odds of depression and an anxiety.

An earlier study has shown that protein-rich dietary pattern can be effective in reducing a number of mental illnesses [38]. Since consumption of BCAAs is a part of a high-protein diet, it seems that high-protein diets can be effective in prevention of psychological disorders. Identification of BCAAs-rich dietary patterns, using reduced-rank regression method, in relation to psychological disorders is suggested.

This study has some strength. This is the first study to examine the relation between BCAAs intake and psychological disorders in human. The large sample size of this study and considering a wide range of potential confounders in statistical analysis are other strengths of this study. However, the current study has some limitations which must be considered. Cross-sectional design of the current study poses limitations in investigating causality. Cross-sectional designs do not allow us to determine whether psychological disorders cause low dietary intakes of BCAAs or if low dietary intakes of BCAAs precedes the development of psychological disorders; therefore, further prospective studies are required to confirm these findings. Moreover, using self-reported data as other limitation might lead to misclassifications of individuals. Although a validated FFQ was used for dietary intakes assessment, the questionnaire was not validated for BCAAs intake. In addition, no information was collected about household income in the current study, which might affect the results.

## Conclusion

In conclusion, an inverse association was found between dietary BCAAs and odds of depression and anxiety. Future large-scale, prospective cohort studies are required to confirm these findings.

## Data Availability

The dataset used and analyzed during the current study is available from the corresponding author on a reasonable request.
